# Medical school selection is a sociohistorical embedded activity: A comparison of five countries

**DOI:** 10.1111/medu.15492

**Published:** 2024-08-09

**Authors:** Jennifer Cleland, Julia Blitz, Eliana Amaral, You You, Kirsty Alexander

**Affiliations:** ^1^ Lee Kong Chian School of Medicine Nanyang Technological University Singapore and National Healthcare Group Singapore Singapore; ^2^ Centre for Health Professions Education, Faculty of Medicine and Health Sciences Stellenbosch University Stellenbosch South Africa; ^3^ Faculdade de Ciências Médicas UNICAMP (Universidade Estadual de Campinas) Campinas São Paulo Brazil; ^4^ Institute of Medical Education/National Center for Health Professions Education Development and Institute of Economics of Education Peking University Beijing China; ^5^ School of Medicine University of Dundee Dundee UK

## Abstract

**Introduction:**

The medical school selection literature comes mostly from a few countries in the Global North and offers little opportunity to consider different ways of thinking and doing. Our aim, therefore, was to critically consider selection practices and their sociohistorical influences in our respective countries (Brazil, China, Singapore, South Africa and the UK), including how any perceived inequalities are addressed.

**Methods:**

This paper summarises many constructive dialogues grounded in the idea of *he er butong* (*和而不同*) (harmony with diversity), learning about and from each other.

**Results:**

Some practices were similar across the five countries, but there were differences in precise practices, attitudes and sociohistorical influences thereon. For example, in Brazil, South Africa and the UK, there is public and political acknowledgement that attainment is linked to systemic and social factors such as socio‐economic status and/or race. Selecting for medical school solely on prior attainment is recognised as unfair to less privileged societal groups. Conversely, selection via examination performance is seen as fair and promoting equality in China and Singapore, although the historical context underpinning this value differs across the two countries. The five countries differ in respect of their actions towards addressing inequality. Quotas are used to ensure the representation of certain groups in Brazil and regional representation in China. Quotas are illegal in the UK, and South Africa does not impose them, leading to the use of various, compensatory ‘workarounds’ to address inequality. Singapore does not take action to address inequality because all people are considered equal constitutionally.

**Discussion:**

In conclusion, medical school selection practices are firmly embedded in history, values, societal expectations and stakeholder beliefs, which vary by context. More comparisons, working from the position of acknowledging and respecting differences, would extend knowledge further and enable consideration of what permits and hinders change in different contexts.

## Abstract 2

Authors from 5 countries offer guidance on how medical school selection reflects the history and values surrounding our institutions in an effort to foster understanding and change.

## 1 INTRODUCTION

As per the mainstream of higher education, medical education research is largely situated within the geographical and epistemological confines of the USA and Western Europe.^1^ Knowledge from countries in South America, Africa and Asia remains peripheral in the geopolitics of knowledge.^2^ Policies, practices and ideals from a few countries in the Global North may be seen as de facto emblematic and globally relevant, and the literature offers little opportunity to consider different ways of thinking and doing. This skewed pattern has implications in terms of how we construct the field of health professions education: what we include or exclude, what we count or not, what we believe to be true or false, what we do or do not read, who speaks and who is silenced.^3^

In our focal area, selection into medical school, an earlier review indicated a near absence of studies from these Global South regions. Patterson et al.^4^ identified only eight articles out of 214 studies from Global South countries, with two studies from Saudi Arabia,^5^, ^6^ four from Pakistan^7^, ^8^, ^9^, ^10^ and one from Thailand^11^ and India.^12^ Indeed, most studies originated from the USA, the UK, Canada and Australia with only an additional four articles from countries outside Europe, North America or Oceania (from Israel, Japan and Taiwan^13^, ^14^, ^15^, ^16^). This tells us that health professions' education researchers in most countries were either not publishing on medical school selection, not publishing in English and/or not publishing in journals listed in mainstream English‐language databases, such as EBSCO, EMBASE, ERIC, Scopus and Web of Knowledge. In short, research on medical school selection reflects the general patterns noted in the literature and offers little opportunity to consider different ways of thinking and doing.

Our ontological position is that of *he er butong* (*和而不同*), a type of universalism that embraces and respects differences.^17^ *He er butong* ‘recognises differences, argues for togetherness, but rejects unification’.^18^, ^43^ Diversity is not only recognised and respected but encouraged and sought after. This position acknowledges that different cultures might have different ways of knowing and valuing evidence and might have different assumptions about what constitutes ‘real’ and ‘important’ knowledge. It helps us understand and practice global or worldwide medical education in a more inclusive and more equitable manner than exists currently, to encompass different perspectives that might shape medical school selection processes in different countries. As per the Chinese philosopher Feng Youlan, ‘the matching of different colours leads to greater beauty’. In short, our position is that we gain by learning about and from each other.

In this contribution of ‘constructive dialogue’ to explore cross‐cultural differences in health professional education, our main aim is to critically consider similarities and differences in approaches to selection and widening access (WA: approaches aimed at increasing the recruitment of certain unrepresented groups into medicine^19^) across our respective countries, of Brazil, China, Singapore, South Africa and the UK. Each country has a different history and legacy. Some were former colonies (Brazil [Portugal], Singapore [UK] and South Africa [the Netherlands and the UK]), one a colonising power (UK) and one (China) avoided being colonised outright (although ceded control of certain areas during the 19th century to various European countries, United States and Japan). Our objective in doing so is to illustrate how, like in education generally, selection practice is a sociohistorical embedded activity.^20^, ^21^ As Taylor et al.^22^ argued, ‘There is always a prior history of significant events, a particular ideological and political climate, a social and economic context’ (p. 16).

As a ‘state of the science’ contribution, this paper does not adhere to the traditional scientific format of introduction, methods, results and discussion. Rather, it represents a summary of many discussions held over time, where, either collectively or in smaller groups, we considered and critiqued selection and widening access practices in our own countries and reflected on underlying socio‐political‐cultural determinants of the parallels and dissimilarities we observed.

## 2 POSITIONALITY

It is important to be explicit about our positionality. Three of us are White, cisgender women with English as a mother tongue (KA, JB and JC). YY is an East Asian woman of Han ethnicity (the ethnic majority in China). EA is a white, cisgender woman with Brazilian Portuguese as a mother tongue. We have education backgrounds in international relations (KA), psychology (JC), economics (YY) and medicine (EA and JB), all currently with research and practice focused on medical education matters. Our primary cultural upbringing and experience with equity issues are within the countries where we are based: Brazil (EA), China (YY), South Africa (JB) and the UK (JC and KA). Some of us (EA, JB, JC and KA) were socialised in white and colonising ways of knowing.^23^ YY did her PhD research overseas but has been mainly socialised in China, a collectivist country with Confucian tradition and a top‐down political system. Although born and raised in the UK, JC now works in Singapore, a country with a divided heritage—one that originates in British colonialism but now tries to represent itself by emphasising its Asian‐ness^24^—where she is part of a cultural minority. JB's earliest experiences were as part of the white colonist community in the British colony of Hong Kong before completing her upbringing in South Africa as part of the white minority (often also viewed as colonist). She holds an awareness that South Africa's apartheid past requires a concerted effort to redress historical issues of access to higher and professional education, particularly in the presence of persistently poor primary and secondary education in many communities. KA works at a university in the context where she was brought up and educated (the UK), bringing the perspective of an academic who is embedded in a global status quo that privileges her. Similarly, EA has always lived in Brazil albeit with short stays in the UK and the USA, working at a state university but constantly interacting and working with international partners and organisations.

We acknowledge that our views are bounded by our experiences of the world^25^: we do not know what we do not know, and we may get things wrong. In the process of writing this paper, we engaged in continual reflexive observations and discussion, consciously trying to identify and reflect on our ways of knowing and doing, to navigate the social landscape and embrace epistemic diversity.

It is also worth mentioning that we are uncomfortable about the terms ‘Global North/South’. However, there is no simple solution, and these terms are less hierarchical and racist than other categorisations of countries.^26^ This classification follows the Brandt Line developed in the 1980s,^27^ and the disparities in relative economic power and income rankings still largely hold today.^28^ As Khan et al.^26^ comment, the classification also indicates ‘the “whiteness” of wealth, including aid, given [Global North] nations are predominately white, while everyone else is not’ (p. 2).

## 3 MEDICAL SCHOOL SELECTION PROCESSES

Before continuing, it is helpful to present a brief overview of popular approaches to medical school selection seen in the published literature (albeit most of this literature is from the Global North: 4). These are prior educational or academic attainment (measured by performance in high school examinations or on an undergraduate [pre‐med] degree in graduate entry medical education; PEA); admissions tests (typically assessing knowledge, general ability and/or aptitude); interviews (either traditional or multiple mini‐interview format); tests of non‐cognitive skills, such as situational judgement or personality tests; personal statements/curricula vitae; and references or letters of recommendation. Many schools use only prior academic attainment in selection decisions. Where more than one tool is used, the sequence of tools is usually as per the following^29^:

- Prior educational attainment, followed by
- an admission/aptitude test, followed by
- some sort of interview.

Widening access (WA) refers to policies and practices at the point of selection, which have been designed and implemented to increase the recruitment of certain unrepresented groups^19^ and thus increase the diversity of medical students and hence doctors. Examples of WA processes and tools reported in the literature include quota systems,^30^, ^31^ outreach programmes, pipeline, gateway and access courses,^32^, ^33^ weighting selection tools^34^ and the use of holistic or contextual data.^35^, ^36^

Table 1 gives some social and economic background to each country and provides a summary of the main approaches to selection and WA to medical school. We had hoped to include data on the representation of different groups in terms of gender, race, class and, where applicable, indigenous status, in this table but were challenged in doing so by the variation in data recency, reliability and availability across the countries under study. Note that in all five countries, medical schools are expected to produce doctors to do postgraduate training and work primarily in state‐funded and controlled health services.

**TABLE 1:** Country and funding context, and main approaches to selection and widening access to medical school in the five countries

Country	Brazil	China	Singapore	South Africa	UK
Population	216 422 446	1 425 671 352	5 920 000	60 414 495	67 736 802
Land mass (km^2^)	8 514 215	9 600 000	750	1 220 813	244 376
Urban/rural inequity in health care delivery	Yes	Yes	No	Yes	Yes
GINI index	52	37	37	63	32
Number of medical students enrolled each year (approx.)	41 800	80 000	450–500	1600	9500
Public (state funded) and/or Private medical schools	Mostly private	Public	Public	Public	Public^a^
First or only tool in medical selection	Performance on the National Exam for High School (ENEM)	Performance on the national college entrance examination (NCEE) or Gaokao (高考) in Chinese	Performance on high school examinations	Performance on high school examinations	Performance on high school examinations
Additional selection tools	University assessments (the vestibular) for entry to public medical schools Multiple Mini interviews used occasionally (but rarely)	N/A	Varies by medical school^b^	Varies by medical school but includes indicators of disadvantage and extracurricular activities	Plus use of selection tests and interviews used by most schools. Personal statements and references used peripherally.
Widening access	Use of quotas for certain under‐represented groups	Use of regional quotas^c^	N/A	Varied use of reserved places for students from rural areas	Reduced academic tariffs Contextual data^d^ Gateway/extended programmes New medical schools focused in areas of patient need

*Note*: The Gini coefficient measures the deviation of the distribution of income (or consumption) among individuals or households within a country from a perfectly equal distribution. A value of 0 represents absolute equality and a value of 100 absolute inequality. Figures reported are from 2023.

^a^ At the time of writing, there was only one private medical school in the UK allowed to award recognised UK medical degrees.

^b^ Singapore has three medical schools, two in partnerships arrangements with a UK and USA medical school, respectively, and which follow the selection practices of those contexts, and one local school.

^c^ The regional quota system of Gaokao does not aim to widen access of underrepresented groups. Rather, it aims to ensure fair opportunity within the province. China does use a separate regional quota for certain underrepresented groups (e.g., students from rural backgrounds). For minority students, the policy gives them additional points for their Gaokao scores, but admission is based on the original regional quota.

^d^ Examples of contextual indicators used in the UK include individual and household level measures (e.g., first generation in higher education), school level measures (e.g., school average performance) and area level measures.^37^

## 4 ATTAINMENT AS PRIMARY SELECTION INDICATOR

Table 1 illustrates that selection into medical school is primarily based on prior educational attainment (PEA) in all five countries. Precisely how PEA is assessed varies—by high school outcomes (Singapore, South Africa and the UK) or knowledge‐based entrance examinations (Brazil and China)—but academic performance/attainment is the common first hurdle into medicine. However, attitudes towards this hurdle, or obligatory passing point,^38^ differ across the countries.

In Brazil, South Africa and the UK, there is public and political acknowledgement that educational attainment is linked to systemic and social factors and hence basing medical school selection on examination performance is recognised as unfair to those who are less privileged in society. Medical school admissions based on PEA effectively maintain social inequalities by being more easily achievable for already privileged levels of society.^39^

Brazil struggles with a post‐colonial legacy of socioeconomic and racial inequalities regarding the access to university.^31^ Since the late 1990s, state governments and the federal government have been working to develop public policies to ensure that the medical student population represents the country's racial and socio‐economic diversity.^40^ In 2012, the Brazilian Supreme Court unanimously deemed that affirmative action was constitutional and necessary to fulfil the constitutional principles of equality.^41^, ^42^ The bylaw supporting the inclusion policy for Black, Brown, quilombolas, disabled people and students who had secondary education in public schools was recently reaffirmed for another 10 years period.^43^ These policies successfully changed the profile of students accessing public higher education and medical school.^31^, ^44^ However, better educated and wealthier students remain better prepared to gain positions in the more competitive and better medical schools, which also happen to be the tuition‐free federal or state supported schools.^45^, ^46^

South Africa has also had to redress colonial legacies. At different points in the history of South Africa, English language, Afrikaans language and—drive by apartheid policies in the 1950s—‘ethnically‐based institutions for Blacks, together with separate universities for Coloureds and Indians’^47^ medical schools were established in the country. These language and ethnicity considerations played out in initial selection criteria. Institutions for White students were privileged in terms of support and funding, whereas those institutions to which Black and other students were assigned were inadequately resourced.^48^ Since the 1996 constitution of the Republic of South Africa, medical schools have had to work to redress apartheid‐era inequities in funding and access and change the racial and gender profile of medical students to be representative of the population. Although there has been progress,^49^, ^50^ change is slow, particularly for Black Africans. This is perhaps at least in part because, despite recognition of the ongoing legacy of apartheid in creating a vast educational attainment gap between the ‘haves’ and the ‘have nots’ in primary and secondary education,^51^ South African medical schools continue to use PEA as the primary hurdle in the selection process.^50^

In the UK, since the 1990s governmental policies have scrutinised the role of PEA in selection to higher education.^52^ In 2012, particular focus fell on medicine, as 80% of medical school applicants came from only 20% of secondary schools.^53^, ^54^ Recognising that high PEA was the major hurdle for selection into medicine, it was thought that policy issued by UK governments^55^, ^56^ might cause schools to reduce emphasis on PEA.^4^ Indeed, the selection processes of UK medical schools have changed substantially in recent years (see later). However, despite these changes, medicine and dentistry still have the lowest representation of students from lower social backgrounds in comparison to other university courses.^57^

In each country, there are long‐standing tensions between the discourses of diversity and excellence stemming from the belief that lowering entry grades for some groups to broaden diversity might result in lower standards and graduation rates.^58^ Addressing that tension is at different stages of maturity in each county, linked to political drivers. For example, in Brazil, affirmative action is legal and constitutional.^41^ In South Africa, despite a government policy (Broad‐Based Black Economic Empowerment) to reverse the apartheid‐era inequalities of participation in the economy, anomalously, there is no imposition on medical schools of a requirement to implement ethnic quotas in selection. In the UK, affirmative action is illegal. However, in the UK, there is nonetheless increasing pressure on universities from governmental policies to increase the diversity of entrants,^54^, ^59^, ^60^, ^61^ including an All‐Parliamentary Group statement^62^ that universities should use contextualise admissions for medicine, which may result in less privileged applicants having a lower PEA requirement (see below).

Interestingly, in Brazil, there are enduring arguments that affirmative action (i.e., accepting people who self‐identify in categories other than White) from public schools of lower socioeconomic status who perform worse on the vestibular exam (the primary and widespread entrance system used by Brazilian universities, from the Portuguese vestíbulo, ‘entrance hall’) and ENEM because of their poor schooling (see Table 1) diminishes and degrades the quality of university education.^63^, ^64^ Nonetheless, more recent studies have shown that affirmative action students perform just as well as those who enter through traditional routes.^44^, ^46^, ^65^, ^66^ As per Brazil, there is growing evidence in the UK that those selected into medicine with lower PEA ‘catch up’ with their peers who entered via traditional routes.^67^, ^68^

The impact of lowering grades for some groups may be more concrete for medical schools themselves in some contexts. In South Africa, for example, all medical faculties are publicly funded (albeit students also pay fees). The subsidy is determined largely by throughput and graduation efficiencies. This funding model may inadvertently discourage medical schools without a very strong social justice mandate from looking beyond the traditional measure of university success, namely, prior educational attainment, as the keystone for selection. This adds to the tension between readdressing historical inequalities and selecting students who have the potential to manage the academic and other demands of studying medicine.

In contrast, in China and Singapore, basing admission to medical school on examination performance continues to be seen as fair and promoting equality. The historical reasons underpinning this belief differ a little across the two countries. In Ancient China, the Imperial Examination was used to select capable candidates for Imperial positions thousands of years ago. The Chinese educational system has remained examination oriented since this time, grounded in a philosophical belief in fairness, which promotes equality based on test performance as opposed to family connections, socio‐economic status and sponsorship.^69^ Although the Imperial Examination is long gone, large‐scale high‐stakes test organised by the central government carries on as a tradition underpinned by the societal belief that the national college entrance examination (NCEE, also known as the Gaokao [高考] in Chinese) is meritocratic^70^ and provides all students with the same starting point.^71^ However, there is acknowledgement of differences in opportunity, evidenced by different Gaokao thresholds for students from rural and/or ethnic minority backgrounds.^72^ Interestingly, recent education reforms to improve the quality of medical education, driven by government policy, have resulted in the selection of students with higher PEA to new programmes—and these students are more likely to come from higher social classes and urban backgrounds.^73^

In the initial years of decolonialisation, Singapore focused on developing a common curriculum for its multi‐lingual population and a national system of high‐stakes and standardised summative exams designed to produce citizens who could contribute to the workforce^74^ and towards Singapore's competitiveness in a globalised capitalist world economy.^75^ The system was based on meritocracy: the educational experience and national assessments were seen to be fair, objective and offering equal opportunities for each student to learn and to achieve his or her potential^75^ and, in doing so, derive socially and economically desirable outcomes. Time may have passed, but there remains a nationwide obsession with aspiration, closely tied to excelling in examinations and reinforced by institutional structures and processes.^76^, ^77^ There is the attitude that less successful individuals have not tried hard enough rather than having fewer social or economic advantages.^78^ Perhaps because of this attitude, the only mechanism for entry for those who do not reach the PEA threshold is excellence in other areas (e.g., sport). The PEA threshold reduction for this ‘aptitude‐based admissions’ category is small, and the process lacks transparency.

Singapore's approach to selection might have been expected to change when new actors came into the system, specifically the establishment of two new medical schools in 2005 and 2013, set up in partnership with overseas universities. These new partners ‘imported’ new norms and attitudes towards selection, specifically the use of additional selection tools (admissions tests and interviews). This disrupted the system, bringing in a new discourse that achievement in high‐stakes national summative assessments is only one indication of capability to study medicine: other qualities, which must be assessed in other ways, are also desirable. However, these new tools were layered onto unchanging systemic, social and cultural attitudes that grades are everything (see earlier), and so these new approaches are, to all intents and purposes, merely a means of selecting between many academically well‐qualified applicants: local estimates indicate that more than half of Singaporean medical students come from the most selective 11% of high schools/junior colleges. This reflects the situation in the UK,^53^ yet there is no public discussion about widening access to medicine in Singapore.

## 5 TO QUOTA OR NOT TO QUOTA?

The need to train doctors to meet population health needs is a common goal across all five contexts. The strength of this mandate is usually linked to the extent of healthcare inequality in the country. For example, there are huge and long‐standing urban–rural or geographical medical workforce disparities and well‐documented imbalances in economic development, health resources and health care between different regions within the vast countries of Brazil, China and South Africa.^79^, ^80^, ^81^, ^82^ One of the big differences we noticed was that in some of the five countries, quotas are used to either ensure that the medical student population represents the country's racial, ethnic and socio‐economic diversity and/or recruit and train doctors to work in underserved areas, and in other countries they are not.

Since the 2012 affirmative action decree (see earlier), all public universities in Brazil must reserve 50% of their places for students coming from public high schools (with the view that students from richer families attend private high schools).^83^ Within this reserved category, there are specific sub‐quotas for different racial groups, indigenous people, people with low income and disabilities. As mentioned earlier, this law was recently reassured, keeping the socio‐economic criteria as primary, but being explicit about achieving quotes in respect of race, indigenous, quilombolas and disabled students for each educational programme.^43^

China has a regional quota system. Each year, colleges and universities draw up enrolment plans and set up a quota for each province. Each province ranks students according to their Gaokao scores. Colleges and universities admit students from each province according to their ranking, ensuring a fair opportunity for all within the province. Interestingly, the admissions criteria in terms of Gaokao scores range widely by medical education programme. For example, in 2023, the minimum admission lines on Gaokao scores for undergraduate education programmes in clinical medicine in different schools varied from about 700 points (the maximum score is 750 points) to 340 points (calculated according to www.gaokao.cn). There are several reasons for this. Medical schools in China are stratified into three tiers linked to schools' funding sources and the types of programmes allowed to be offered.^84^ Higher tiered medical programmes set higher Gaokao thresholds, lower tier and remote and rural medical programmes set lower thresholds.

Three of our five countries do not use quota systems. Academic freedom (including institutional autonomy) is enshrined in the 1996 constitution of the Republic of South Africa enabling the fulfilment of higher education's accountability to society. It may well be this constitutional aim that has, so far, prevented any requirement for medical schools to implement ethnicity‐based quotas in selection. In some ways, this appears to be an anomaly in a country that adopted a government policy to reverse the apartheid era inequalities in broad participation in the economy. In the UK, any discrimination, quotas or favouritism due to sex, race and ethnicity among other ‘protected characteristics’ is illegal by default in education and other areas, as per the UK's Equality Act 2010.

In both countries, in the absence of quotas, different strategies such as introducing tools additional to PEA into the selection process have been adopted to fulfil the same aim of increasing the diversity of medical students.^50^, ^60^ Common across both countries is taking indicators of disadvantage such as family income, schooling and rural origin (contextual data) into account when making selection decisions (see also Table 1). Indeed, 90% of UK medical schools used contextual admissions (CA) for entry in 2024,^37^ and nearly all also utilise an interview and admissions test.^85^ Key stakeholders (e.g., students, teachers, interest groups and government) seem generally supportive,^86^ but a substantial minority oppose or have significant concerns about the use of CA, which varies substantially between schools.^87^ Similarly, in South Africa, each medical school employs a particular combination which may include measures of disadvantage (extent of poverty in the community where their secondary school was located, family receiving a social grant, parental education level), indicators of rural origin, interviews, and so on, but currently, there is no uniformity in how they are applied across the medical schools.^50^ Full consideration of the success or otherwise of these approaches, or indeed of quotas, is beyond the scope of this paper.

Singapore, on the other hand, has neither a quota system—the former Prime Minister Lee Hsien Loong stated that ‘we [Singapore] have no affirmative action or quota system here in any field of endeavour’^88^—nor does it use CA other than as described earlier. The belief is, or has been, that such measures are not necessary because Singapore is constitutionally an equal society, with high social mobility linked to economic transformation since decolonialisation. However, although Singapore's GINI coefficient is relatively low, perceptions of inequality and social stratification are rising, as is evidence of educational inequality.^89^, ^90^, ^91^ Discussions about how to increase the diversity of medical students in Singapore may not be so far away.

Interestingly, of the five countries considered in this paper, the two with quota systems, China and Brazil, have the most complete and most easily accessible data on their medical student demographics.^73^, ^83^ This may be related to the fact that institutions in these countries are scrutinised for their outcomes in terms of achieving quotas.

## 6 DISCUSSION

In this constructive dialogue, we critically considered approaches to selection and widening access to medical school across different countries. We stepped beyond the North–South dialogue to a more nuanced, democratic consideration of the contextual drivers of medical school selection in four Global South (Brazil, China, Singapore and South Africa) and one Global North (UK) country. We must admit to initially assuming Singapore was Global North given it is categorised as a developed and high‐income country, illustrating the limitations of categorisation.^26^

We hope to have achieved our aim to have provided insight into how approaches to medical school selection are firmly embedded in tradition and history, norms and values, societal expectations and stakeholder beliefs, which vary by context. There were, however, some general messages. Change tends to be gradual (if it happens at all), driven by politics and policy goals. Policy‐influenced changes in practice are nearly always cumulatively added over time, layered on what came before and what is allowed and acceptable, which can result in complexities, tensions and contradictions in systems.^92^ Given this, what works in one place or at one time is unlikely to work in the same way in another place or at another time.

The process of writing this paper revealed the large extent to which three of us, as researchers, are embedded in the academic norms and power structures determined by and benefitting the global north (JB, JC and KA) whereas YY and EA have slightly different positions and understanding. Our discussions illuminated to us how, in the everyday, we often unquestionably looked to evidence and practices from a familiar range of countries, structured on familiar concepts and presented in familiar language, whether this be English, Brazilian Portuguese or Chinese. Our ontological position, *he er butong* (*和而不同*)^17^, ^18^ challenged us to think curiously and critically, and to expose and challenge dismissive and judgemental thoughts. Accepting that there are different ways of valuing evidence and that we can be ‘together’ in discussions about selection without being unified in our approaches and conclusions underpinned this piece.

Upon reflection, we also see that how our comparison was unconsciously (and thus perhaps inevitably) shaped by power dynamics and connections within the field. In their description of the Global South, Khan et al.^26^ note: ‘there are many countries in the South, that are increasingly at par with rich Northern countries, like India, China and Brazil’ (p. 2). These were some of the countries that we, as authors, belong to and choose to explore as our Global South contexts. We call for further comparative papers of this nature to bring more voices into the discussion.

Ironically, in our quest to acknowledge and respect differences, we reinforced inequity by depending on English as our lingua franca. Although a comprehensive exploration of English‐language imperialism, its inequitable ‘othering’^93^, ^94^ of colleagues who are non‐native English‐speakers and its effects in academia and publishing is beyond the scope of this paper, we urge colleagues to consider the marginalisation of other languages, limiting access to knowledge published in languages other than English, and how this might influence ways of thinking.

Working together may have illuminated our personal limitations but it also reminded us of how important it is to look beyond our bounded domains of not just our own contexts but also health professions education, to expand our knowledge and question our assumptions. We read many economic and political papers about the Global North–South divide to address our curiosity about different classification systems. We also realised the importance of papers giving sufficient details about context irrespective of where in the globe their claims are produced. To do so is critical to meaning and generalisability.

We feel that we have only ‘scratched the surface’ in this paper. Our constructive dialogues were much more wide‐ranging than can be encompassed in 4000 words or thereabouts. We intend to continue our discussions, to understand our differences and similarities and to continue to learn about and from each other.

## 7 CONCLUSION

To shift from the hegemony of the Global North to multipolarity of knowledge and perspectives means that, rather than trying to take facts out of their contexts to draw universal conclusions from them, we need to situate selection practices and associated research within their context, being aware of the historical, social and economic processes, conditions, forms and effects of the dynamics which influence context.^95^ Acknowledging the independent socially and historically embedded identities of countries encourages learning from differences and similarities and opens the door to more comparative research, which is mostly lacking in this area.^38^, ^96^, ^97^ More comparisons, working from the position of acknowledging and respecting differences, would extend knowledge, and open space to bring in different ontologies/epistemologies/axiologies/methods/theories in this area, as well as examining what permits and hinders changes in different contexts.

## ETHICS STATEMENT

This paper reports a review of previously published research. No human subjects were involved in data collection.

## AUTHOR CONTRIBUTIONS

Julia Blitz, Jennifer Cleland, Kirsty Alexander and You You conceptualised the work. Julia Blitz, Jennifer Cleland, Kirsty Alexander and You You conceptualised the work. All authors were involved in dialogues by email and via teleconferencing. Jennifer Cleland prepared the drafts of the manuscript, which were developed over time with inputs from Julia Blitz, Kirsty Alexander, Eliana Amaral and You You. All authors reviewed a near final version of the manuscript, gave approval for publication and agree to be accountable for the work.

## CONFLICT OF INTEREST STATEMENT

We declare no competing interest.

## Data Availability

Data sharing is not applicable to this article, as no new data were created or analysed in this study.
